# Phenotypes of juvenile idiopathic arthritis

**DOI:** 10.1186/1546-0096-12-S1-P161

**Published:** 2014-09-17

**Authors:** Faina Rokhlina, Gennadiy Novik

**Affiliations:** 1 SPbGPMU, St.Petersburg, Russian Federation

## Introduction

The term "phenotype" comes from the Greek word "phaino" - are - is the mix of characteristics and properties of the body, formed in the process of individual development. In medicine phenotype has traditionally been viewed as the result of the interaction of the genotype (genetic characteristics of the organism to the conditions of external environment. Different authors in the allocation of individual phenotypes describe clinical and morphological characteristics of the disease, the most significant triggers, the presence of the leading link of the pathogenesis of the disease, as well as a unique response to treatment.

## Objectives

The aim of our study was to learn structural (polymorphism C3435T) and functional (P-glycoprotein) features MDR1гена in children with JIA.

## Methods

The relative number of peripheral blood lymphocytes expressing his membrane MDR-receptor (P-glycoprotein), was determined by flow cytometry.

The definition of polymorphism C3435T MDR1 gene. Amplification was performed on the device “ICycler (BioRad, USA) by standard scheme.

Determination of cytokines (IL1, IL6, TNF-alpha) in the serum of patients with JIA was conducted by the method of solid-phase enzyme immunoassay.

Determining the concentration of methotrexate in the blood serum were conducted on the analyzer TDxFLx firm Abbott method fluorescently-polarization immunoassay.

## Results

For MDR1-dependent phenotype characterized by a higher activity of the disease, high levels TNFα and IL6. This phenotype is more common in patients with genotype CT and CC of polymorphism C3435T MDR1 gene, have higher relative amount of lymphocytes expressing P-glycoprotein as to stimulate IL2 and after; low MTX concentration in the serum.

MDR1-independent phenotype characterized by lower activity of the disease, low content TNFα and IL6 in the serum, the most common genotype TT polymorphism C3435T MDR1 gene. Characterized by a relatively low number of lymphocytes expressing P-glycoprotein as to stimulate IL2 and after, and higher values MTX concentration in the serum.

## Conclusion

After analyzing the obtained results we can assume that the study of the MDR1 gene polymorphism is necessary to assess the impact of different mechanisms on the concentration of methotrexate in patients with JIA, provides the ability to simulate dose methotrexate depending on the response to therapy. So, in patients with MDR-dependent phenotype can be considered earlier appointment of genetically engineered biological agents.

The selection of phenotypes diseases can afford to individualize the therapy to achieve better control of the disease.

## Disclosure of interest

None declared.

